# A prospective non-randomized controlled trial testing the effectiveness of psychotherapeutic inpatient treatment of Post-COVID-19 syndrome - study protocol

**DOI:** 10.1186/s40359-024-01974-5

**Published:** 2024-09-16

**Authors:** Katharina Koller, Silke Kastel-Hoffmann, Regina Herold, Eva Morawa, Marietta Lieb, Johannes Krehbiel, Bettina Hohberger, Yesim Erim

**Affiliations:** 1 Department of Psychosomatic Medicine and Psychotherapy, Universitätsklinikum Erlangen, Friedrich- Alexander-Universität Erlangen-Nürnberg (FAU), Erlangen, Germany; 2 Post-COVID Center, Universitätsklinikum Erlangen, Erlangen, Germany; 3 Department of Ophthalmology, Universitätsklinikum Erlangen, Friedrich-Alexander-Universität Erlangen- Nürnberg (FAU), Erlangen, Germany

**Keywords:** COVID-19, Post-COVID syndrome, Fatigue, Mental illness, Psychotherapy, Coping with illness

## Abstract

**Introduction:**

In addition to physical symptoms such as dyspnea, fatigue, post-exertional malaise, and pain, a subgroup of patients with Post-COVID-19 syndrome (Post-Acute Sequelae of COVID-19, PASC) suffers from mental illnesses such as anxiety, depression, and neurocognitive impairments. To date, there are no causal treatments available for PASC. While initial studies show that psychotherapy improves psychological symptoms, PASC-related fatigue, and psychosocial functioning, further research is needed to evaluate the effectiveness of psychotherapeutic treatment for PASC.

**Methods and analysis:**

This study presents a non-randomized controlled trial aimed at evaluating the effectiveness of a five-week multimodal inpatient psychosomatic treatment program for individuals experiencing PASC symptoms and comorbid mental illness. A total of 118 patients presented at the Post-COVID Center at the Universitätsklinikum Erlangen will be assigned to the intervention group receiving inpatient psychosomatic treatment or the control group receiving treatment as usual. The inclusion criteria for the intervention group are a diagnosis of PASC and at least one condition of mental distress and problems with coping with illness. The primary objective of the intervention is to reduce mental ailments, including depression and anxiety, as well as neurocognitive deficits, and to address PASC symptoms such as fatigue and pain. The core elements of the treatment are psychotherapy in individual and group settings, medical treatment, neurocognitive training, and physical therapy, adapted to the individual’s capacity and oriented towards the concept of pacing. After enrollment, participants will undergo a 6-month follow-up to assess long-term results and the sustainability of the intervention effects.

**Discussion:**

This study examines the effectiveness of inpatient psychotherapeutic treatment in PASC patients with comorbid mental illness in comparison with a control group based on treatment as usual. The results of the study can contribute to the development of evidence-based interventions to address the complex needs of patients with PASC and comorbid mental illness.

**Trial registration:**

German Clinical Trial Register (DRKS), retrospectively registered 15.02.2024 DRKSID DRKS00033562.

**Supplementary Information:**

The online version contains supplementary material available at 10.1186/s40359-024-01974-5.

## Introduction

The World Health Organization (WHO) characterizes Post-COVID-19 syndrome (Post-Acute Sequelae of COVID-19, PASC) as a condition where new or ongoing symptoms arise three months after the initial SARS-CoV-2 infection, lasting for a minimum of twelve weeks and lacking an alternative explanation [1, 2]. Among those infected with SARS-CoV-2, 6.2% experience at least one of three PASC symptom clusters, which include persistent fatigue with bodily pain or psychological problems, neurocognitive problems, or ongoing respiratory issues. Prevalence rates of PASC vary between different studies and subgroups, with the highest rates among women and those who required hospitalization for the initial SARS-CoV-2 infection [3–5]. Worldwide, physicians face considerable difficulties in treating PASC due to the absence of specific biological markers for clear diagnostic classification and the lack of causal therapies, complicating effective treatment of PASC and raising economic costs due to frequent contact with general practitioners and specialist physicians [6]. For those affected, PASC not only significantly impairs their quality of life [7], but also their ability to work. In 2022 alone, the German Pension Insurance reported 21,000 inpatient rehabilitation treatments for PASC [8] and 1,000 approved pensions for those unable to return to work due to the condition [9].

Approximately one-fifth of PASC patients also reveal mental health comorbidities such as anxiety, depression, symptoms of PTSD, and disruptions in sleep patterns [10, 11], with a prevalence of depressive and anxiety symptoms 12 months after SARS-CoV-2 infection at 23% and 22%, respectively. The prevalence of insomnia was found to be 12% [12], while other studies estimated sleep disturbances to be as high as 45% [13]. Posttraumatic symptoms after SARS-CoV-2 infection range from 6.5 to 42.8%, particularly high in those who had severe SARS-CoV-2 infection [14]. There is also accumulating evidence that preexisting psychological and psychosomatic conditions, such as symptoms of depression and anxiety, loneliness, and perceived stress, are vulnerability factors for the appearance of PASC symptoms [15]. Notably, depressive symptoms along with anxiety symptoms were found to persist for an extended period [16–18] and can significantly impact an individual’s quality of life [7].

One possible explanation for the occurrence of accompanying mental illness in PASC patients may be the difficulties in coping with illness, a concept explained by the stress-coping model [19]. The onset of a chronic illness is typically associated with psychosocial stress arising from e.g. physical limitations, the unpredictability of the disease’s progression, threats to social roles and activities, financial problems, and disruptions in self-concept and emotional well-being [20]. Effective stress management depends on evaluating stressors and having adequate coping resources, e.g. psychological stability or social support [19]. When stress exceeds these resources, the likelihood of mental illness increases [21, 22].

At the same time, psychosocial factors, in addition to biomedical factors, play an important role in maintaining and exacerbating persistent physical symptoms, defined as distressing somatic complaints lasting several months or longer. Psychosocial factors can include cognitive-perceptual and emotional mechanisms such as symptom focusing, catastrophizing interpretations, and disease-related fears or behavioral processes such as avoidance behavior, physical inactivity, and deconditioning [23]. This interplay of biomedical and psychosocial factors is also discussed for the persistence of physical symptoms in PASC [24, 25]. An initial study showed that psychological distress associated with persistent symptoms that occurred during the first year of the pandemic predicted the presence of at least one persistent symptom 6–10 months later [26]. This finding underscores the possible role of psychosocial stress in the disease course of PASC.

Initial studies have shown positive effects of psychotherapeutic interventions in PASC in outpatient and rehabilitative settings. Cognitive behavioral interventions applied in an outpatient setting lead to an improvement in fatigue symptoms and improved coping in PASC patients [27, 28]. Multimodal inpatient rehabilitation treatment with psychotherapeutic elements showed a reduction in psychological and somatic symptoms and an improvement in activity and social participation [29]. However, while psychotherapeutic interventions for PASC have been evaluated in outpatient and rehabilitation settings, results on the effectiveness of psychotherapeutic interventions established in acute care of psychosomatic treatment are lacking. When we refer to “psychosomatic treatment” in the following, we mean inpatient multimodal, focus-orientated psychotherapy for the treatment of PASC patients with mental disorders.

To address mental disorders and coping with PASC symptoms, we will use psychotherapeutic interventions which include e.g., psycho-education, coping with fear of progression, working on resources, relaxation techniques, working on emotions, and cognitive methods such as cognitive restructuring [30]. These approaches are already used as interventions for chronic illnesses, e.g. in psycho-oncological care and rehabilitation of chronic diseases, and can help patients manage their illness more effectively [31, 32]. To treat more physical symptoms of PASC, such as breathing problems and fatigue, we will supplement psychotherapeutic interventions with physiotherapy. Research on other chronic diseases, such as multiple sclerosis, has demonstrated the beneficial effects of aerobic exercises on fatigue symptoms [33, 34]. Furthermore, aerobic exercise was shown to be effective and safe as an early intervention after (severe) SARS-CoV-2 infection [35] as well as a treatment to improve PASC-related fatigue and functional capacity [29, 36, 37]. When combined with the concept of pacing - a self-management strategy that involves tailoring physical and cognitive activity to the individual’s energy levels [2]- aerobic exercise can be safely incorporated without risking post-exertional malaise (PEM), a worsening of chronic fatigue symptoms after minimal physical or mental exertion [38]. Various studies have shown that activity pacing positively impacts fatigue severity in chronic diseases [39–41]. Aerobic exercise can also improve neurocognitive functioning, which has been shown in a rehabilitation exercise program that incorporates resistance, endurance, and balance training, as well as education and pacing strategies [36]. To further alleviate the neurocognitive impairments, we will use computerized neurocognitive training, which has shown promising results in PASC patients according to initial studies. [42].

In order to appropriately tackle all these issues of PASC, an integrated multimodal approach in a psychosomatic treatment setting is ideal. In our trial, we are testing this inpatient psychosomatic PASC setting for effectiveness with a control group. Key endpoints include changes in the severity of PASC (primary outcome), as well as overall physical and mental health symptoms such as insomnia, fatigue, PEM, neurocognitive impairments, anxiety, and depression (secondary outcomes). Additional secondary outcome measures include perceived social support, distress, sense of coherence, quality of life, and coping with illness. Moreover, outcomes like healthcare utilization and treatment satisfaction are assessed.

### Objectives and hypothesis

The objective of the proposed trial is to address PASC symptoms and provide an effective treatment for PASC patients with accompanying mental illness. We aim to assess the effectiveness of a five-week inpatient treatment in the Department of Psychosomatic Medicine and Psychotherapy at Universitätsklinikum Erlangen.

### Primary hypothesis

We expect the intervention to be superior to treatment as usual (TAU) regarding a significantly lower mean of PASC symptoms measured by the Post-COVID Syndrome Scale (PCS Score) post-treatment and at 6 months follow-up.

### Secondary hypothesis

We expect the intervention to be superior to TAU in terms of reducing depression and anxiety symptoms, fatigue, sleeping problems, and perceived stress that relates to the illness as well as improving neurocognitive functions, patient competence to cope with illness, perceived social support, overall quality of life, and sense of coherence.

## Methods

For reporting the outcomes of inpatient psychosomatic treatment for PASC, the SPIRIT (Standard Protocol Items: Recommendations for Interventional Trials) reporting guideline will be used [43]. In case of important protocol modification, it will be reported to the sponsor and the ethics committee, and the trial registration will be updated. Results will be published in peer-reviewed journals, conference presentations, and among self-help organizations.

### Study design

**Fig. 1:**
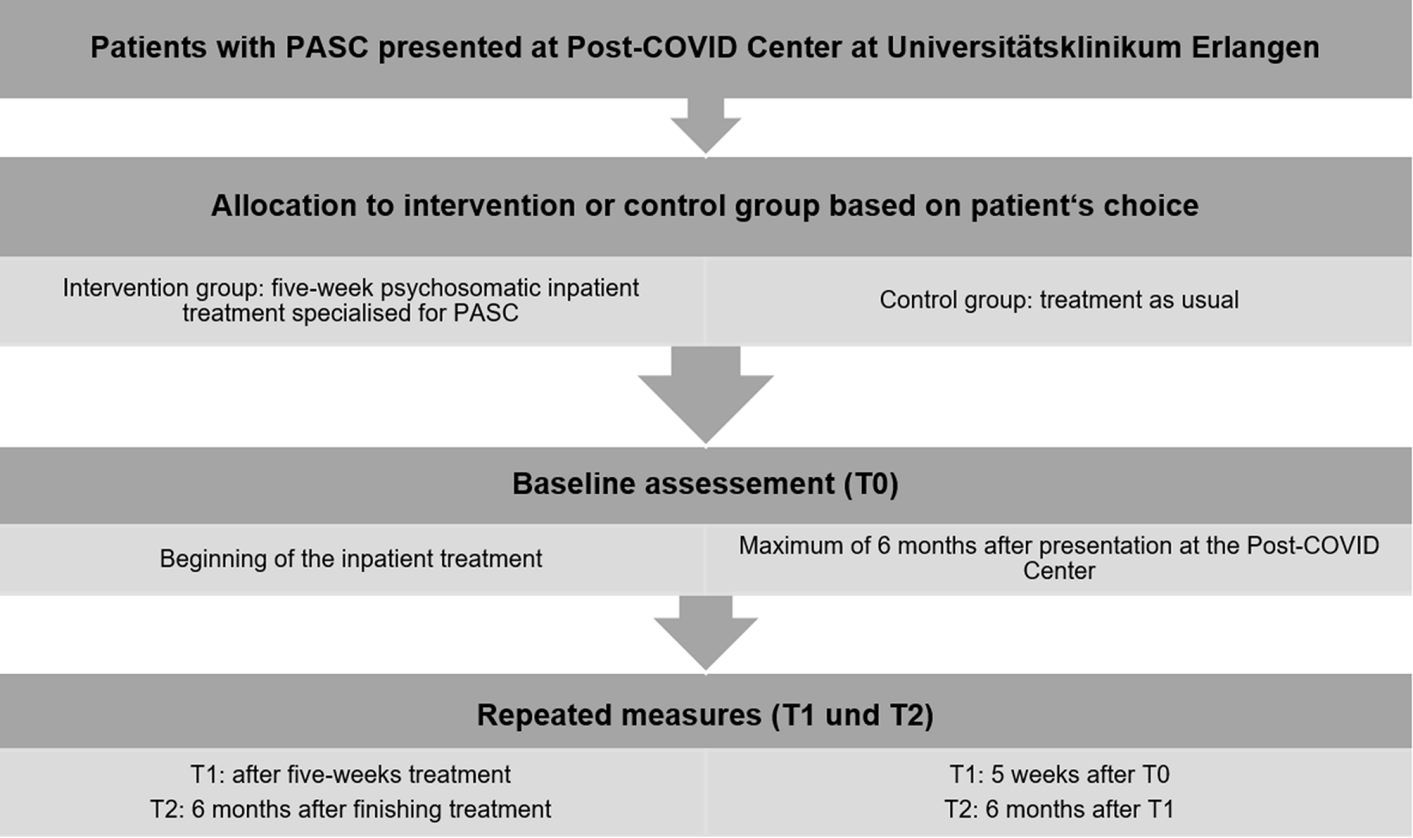
Trial flow

This is a prospective non-randomized controlled trial. It is a two (group: intervention and control) by three (time: pre-treatment, 5 weeks, 6 months) repeated measures factorial design. The overall study design is illustrated in Fig. 1 and Table 1. In T0 sociodemographic data, medication, and use of previous psychotherapeutic inpatient or outpatient treatments, as well as data on health behavior (e.g. smoking, alcohol consumption, etc.) of the patients will be collected. Participants in the intervention group will complete a comprehensive web-based questionnaire battery at three time points: at the time of admission to the hospital setting (T0), at the end of inpatient treatment five weeks later (T1), and 6 months after discharge from the hospital setting (T2). The control group will accomplish the questionnaire within a time frame of around 6 months after presentation at the Post-COVID Center of the Universitätsklinikum Erlangen (T0), five weeks after T0 (T1), and 6 months after T0 (T2). The questionnaires at T0, T1, and T2 will be made available web-based via a link and a password. Participants who drop out of the intervention or control group will be reported in the final results. Before participation, written informed consent must be given. A schematic overview of the trial flow is given in Fig. 1. The instruments used at each time point are displayed in Table 1.

**Table 1:** Overview of the assessment schedule for both groups

Measures	Assessment time points		
	Baseline (T0)	5 weeks later (T1)	6-month follow-up (T2)
Sociodemographic variables	X		
Health behavior	X	X	X
Use of PASC-specific treatments		X**	X
Use of PASC-specific outpatient or inpatient psychotherapeutic treatment	X	X**	X
**Primary outcome**			
PASC (PCS Score)	X	X	X
**Secondary outcomes**			
Somatic, Depression, and Anxiety symptoms (PHQ-15; PHQ-9; GAD–7)	X	X	X
Sleep (ISI)	X	X	X
Fatigue (FSS)	X	X	X
Post-exertional malaise (PEM)	X	X	X
Social support (ESSI)	X	X	X
Sense of coherence (SOC-3)	X	X	X
Distress (PSS-4)	X	X	X
Somatic symptom disorder (SSD-12)	X	X	X
Patients’ competence to cope with illness (PUK-2)	X	X	X
Quality of life (SF-36)	X	X	X
Satisfaction with treatment			X*
Neurocognitive testing*			
Declarative verbal memory (retrieval and recognition of VLMT)	X	X	
Cognitive processing speed and cognitive flexibility (TMT)	X	X	
Numerical short-term memory and working memory (Digit span backwards of WMS – R)	X	X	
Attention and concentration (d2 – R)	X	X	
Formal lexical and semantic verbal fluency (RWT)	X	X	

* Only for the intervention group

** Only for the control group

PCS Score, Post-COVID Score; PHQ-15, Patient Health Questionnaire-15; PHQ-9, Patient Health Questionnaire-9; GAD – 7, Generalized Anxiety Disorder Scale – 7; ISI, Insomnia Severity Index; FSS, Fatigue Severity Scale; PEM, Post-Exertional Malaise; ESSI, ENRICHD Social Support Instrument; SOC-3, Sense of Coherence Scale-3; PSS-4, Perceived Stress Scale-4; SSD-12, Somatic Symptom Disorder-12; PUK-2, Patient Competence in Coping with Cancer Questionnaire adapted for PASC symptoms; SF-36, Short Form Health Survey Questionnaire; VLMT, Verbal Learning and Memory Test; TMT, Trail Making Test; WMS-R, Wechsler Memory Scale-R; d2-R, Attention and Concentration Test; RWT, Regensburger Verbal Fluency Test

### Participant’s eligibility and recruitment

Patients will be recruited from the Post-COVID Center at the Universitätsklinikum Erlangen. All study patients will receive comprehensive interdisciplinary diagnostics at the Post-COVID Center. After detailed information and consent, eligible patients will be allocated to either a control or intervention group.

#### Inclusion criteria

1. Aged 18 years or older.
2. Confirmed (e.g. positive PCR or antigen test) SARS-CoV-2 infection and subsequent PASC: complaints that are still present more than 12 weeks after the onset of SARS-CoV-2 infection and cannot be explained otherwise.
3. Written informed consent to participate in the study.
4. Sufficient knowledge of the German language to participate in the study.
5. Treatment group: indication for inpatient psychosomatic treatment due to:

- Diagnosis of mental disorder (ICD-10; International Classification of Diseases, 10th Revision).
- Limitations that significantly reduce the ability to manage everyday life (e.g. the ability to maintain daily structure).
  and/ or

- Subjective stress so great that the quality of life and everyday resilience are significantly limited.

6) Control group: depressive symptoms in self-assessment (PHQ-9 ≥ 10).

#### Exclusion criteria

1. Severely physically impaired patients (predominantly bedridden).
2. Unique or main diagnosis of substance abuse (F10 – F19), schizophrenia, psychosis (F20 – F229) or organic psychiatric disorders (F00 – F09).
3. Suicidality.
4. Control group: utilization of PASC-specific outpatient or inpatient psychotherapeutic treatment or psychosomatic rehabilitation during 5 weeks of treatment.

### Control group

The control group will consist of PASC patients for whom there is an indication for inpatient psychosomatic treatment (depressive symptoms in self-assessment: PHQ-9 ≥ 10), but who do not take up the treatment. After group allocation, participants in the TAU condition will have no access to the inpatient psychosomatic treatment, but will not be restrained from using any form of care for PASC-related symptoms. Treatment as usual for PASC patients can entail follow-up contacts with their treating physician or general practitioner, physical training, occupational therapy, or rehabilitation. At T0, T1, and T2, participants of the control group will be questioned about the received psychotherapeutic inpatient or outpatient treatment.

### Intervention

The intervention consists of a five-week inpatient psychosomatic treatment according to an integrative comprehensive concept of depth psychological and cognitive-behavioral therapy. The patients will be admitted in closed groups of 5 to 6 patients each.

All therapists will be physicians in advanced or completed specialized training in psychosomatic medicine and psychologists in advanced or completed training in clinical psychotherapy, physiotherapists, registered nurses, social workers, and psychotherapists for integrative body and movement therapy. At the beginning of the study, all therapists will receive several training courses on psychosomatic aspects of PASC, the PASC guideline, the PASC concept for inpatient psychotherapy, immunological principles of PASC and fatigue, and the treatment of PASC in physiotherapy and aerobic exercise therapy.

**Fig. 2:**
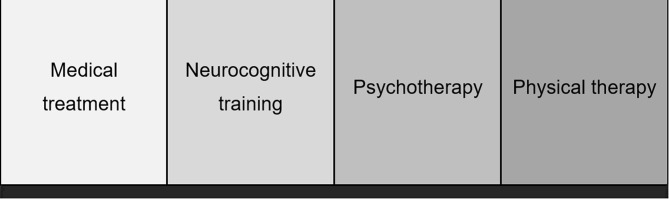
Four cornerstones of PASC specific inpatient psychosomatic treatment

We have adopted parts of the concept from the working group of Köllner et al. [44] and adapted it to our psychosomatic inpatient setting. The psychotherapeutic focus of the treatment is on coping with the PASC symptoms and treating comorbid mental symptoms that have arisen as a result of the chronic illness. Table 2 provides an overview of the treatment interventions, addressing the key aims of each element. Four cornerstones of treatment are applied in addition to our regular multimodal, focus-orientated psychotherapy concept. Figure 2 illustrates these cornerstones, which are explained in detail below.

1. **Medical treatment** Our comprehensive multidisciplinary medical assessment will include detailed medical examination at the beginning and end of treatment, with evaluations by specialists if new symptoms arise and weekly medical consultations.
2. **Neurocognitive training** Neurocognitive training will be carried out independently with the digital health application NeuroNation MED [45] at least twice a week. The individually customized training plan and the adaptive difficulty level will be tailored to the user’s needs. NeuroNation MED contains over 35 multimodal exercises that target the domains of memory, attention, processing speed, and reasoning. The neurocognitive training will be continued after discharge from inpatient treatment. This is made possible because NeuroNation MED is carried out on a smartphone.
3. **Psychotherapy** Our therapeutic approach is characterized by a permanent validation of the psychological symptoms resulting from a somatic illness, in this case, SARS-CoV-2 infection, acceptance of the status quo and at the same time strengthening self-efficacy and resource orientation. The focus of our psychotherapy concept is on developing an understanding of PASC from a biopsychosocial perspective and learning functional coping skills, taking into account the physical, psychological, and social challenges of PASC. The treatment is based on established, effective methods of cognitive behavioral therapy (CBT) and acceptance and commitment therapy (ACT). To avoid post-exertional malaise (PEM) the focus of psychoeducation is on learning the 3P principle [46], including pacing, planning, and prioritizing. Pacing means, for example, dividing tasks and activities into smaller sections throughout the day and taking breaks. Planning refers to the scheduling of support and the prior provision of aids for activities while prioritizing means setting realistic goals and assessing the importance and necessity of various tasks [2]. To ensure pacing, therapies are individually tailored, meaning patients will be allowed to skip therapies at any time as required or cancel them early. This serves as a training ground for recognizing and setting one’s boundaries.
4. **Physical therapy** will take place in close cooperation and coordination with the Department of Physical and Rehabilitative Medicine at the Universitätsklinikum Erlangen. There will be weekly interdisciplinary meetings to discuss the patient’s current status. The so-called CoFit Training Group (e.g. circuit training, stretching exercises, balance training, breathing therapy, back exercises, etc.) will take place in a group setting twice a week for 30 min each. When indicated, patients will also receive additional pulse-controlled ergometer training in a group setting, depending on their fitness level. Our physiotherapeutic treatment concept for post-COVID will take up established physiotherapeutic treatment approaches for fatigue (e.g. for multiple sclerosis) [33, 34] and will include pacing as a central technique in all therapies. Additionally, patients will rate their symptoms and their exertion rate on the Borg breathlessness scale [47]. This ensures that individual resilience will be taken into account in the CoFit training group and ergometer training and that physiotherapy will continuously be adapted to individual’s progress.

**Table 2:** Overview of the frequency and duration, and key aims of psychosomatic treatment for PASC

	Frequency and duration	Key aims
**Psychotherapeutic interventions**		
PASC psychoeducation group	2x/ week for 90 min	Psychoeducation and development of an individualized disorder model; dealing with PASC, changes in lifestyle and self-esteem; communicating about PASC, coping skills, and psychological mechanisms
Individual psychotherapy	2x/ week for 50 min and 25 min	
Family meeting	1 × 60 min when indicated	Involving the social environment to strengthen social support and give information on PASC
Mindfulness walking group	2x/ week for 30 min	Promoting the recognition of physical limits following the concept of pacing, self-expression, and introspection and reducing distress
Relaxation training group (progressive muscular relaxation and yoga)	2x/ week for 30 min	
Mindfulness training group	2x/ week for 30 min	
Integrative body and movement therapy group	1x/ week for 90 min	Improving patients’ self-awareness and body awareness, helping patients accept their current state, promoting self-efficacy and self-care
**Neurocognitive training**		
Neurocognitive training	2x/ week for 30 min	Improving patients’ neurocognitive impairments by individually tailored exercises on the digital health application NeuroNation MED
**Medical treatment**		
Medical consultation hour	1x/ week for 20 min and when indicated	Addressing medical problems, answering medical questions, if necessary improve adherence to intervention protocols
**Physiotherapy**		
CoFit-Training Group	2x/ week for 30 min	Individualized aerobic exercise training to improve physical and mental resilience, reduce oxidative stress, and exhibit positive effects on immune responses
Individual physiotherapy	When indicated	
Ergometer training group	When indicated	
**Additional elements**		
Social worker	1 × 60 min	Socio-therapeutic counseling on social security issues and work-related problems
PASC group visitation	1x/ week for 30 min	Giving and receiving feedback on the course of treatment, coordination, and adjustment of the treatment plan, if necessary improve adherence to intervention protocols

### Outcomes

#### Primary outcome

The primary outcome are symptoms of PASC at the end of the treatment (T1, see Table 1). To measure those, we will use the PCS-Score. The PCS-Score consists of 12 yes/no questions and covers the entire spectrum of possible PASC symptoms. Each question is given its weighting factor, which assigns a meaning to the corresponding symptom complex. The resulting sum score ranges from 0 (= complete absence of any symptoms) to 59 (= presence of symptoms in each of the 12 symptom complexes). A clinically relevant PASC can be assumed from a PCS Score of 26.25 (high PCS Score). Values between 10.75 and 26.25 indicate an intermediate or moderate PASC. The PCS Score was validated on a German population [48].

#### Main secondary outcomes

Somatic, depressive, and anxiety symptoms will be evaluated using the German version of the Patient Health Questionnaire Somatic Symptom Scale 15 (PHQ-15), the Patient Health Questionnaire Depression Scale 9 (PHQ-9), and the Generalized Anxiety Disorder Scale-7 (GAD-7) [49–51]. To assess fatigue, we will use the Fatigue Severity Scale (FSS) [52]. Post-exertional malaise will be measured by an adapted German version of the DePaul Post-Exertional Malaise Questionnaire (PEM). The five DSQ PEM items assess the frequency and severity of PEM over a six-month timeframe. Five additional PEM items examine the duration of symptom exacerbation after activity (3 items), whether patients perceived physical and cognitive exhaustion, and whether participants were not exercising because it made their symptoms worse [53, 54]. Sleep disturbances will be quantified using the Insomnia Severity Index (ISI), a validated measure of insomnia severity and its impact on daily functioning [55, 56]. Social support, a key factor in coping with illness and adversity, will be assessed using a validated German version of the ENRICHD Social Support Instrument (ESSI), which captures perceived social support [57]. The short version of the Sense of Coherence Scale (SOC-3) will be used to assess the participants’ perceived ability to cope with stressors and maintain a sense of coherence and meaning in their lives [58]. Perceived stress levels will be measured using the Perceived Stress Scale (PSS-4), a short version of the 14-item Perceived Stress Scale, a widely used instrument for assessing the degree to which situations in one’s life are appraised as stressful [59]. Furthermore, psychological stress specific to somatic symptoms disorder (SSD-12) will be evaluated to capture the stress experienced in response to physical symptoms [60]. Participants’ coping strategies in response to PASC symptoms will be evaluated using the Patient Competence in Coping with Cancer Questionnaire (PUK-2) adapted for PASC symptoms. This measure aims to assess the perceived competence of participants to manage and cope with the challenges posed by ongoing symptoms [61]. Finally, patients’ health-related quality of life will be measured by using the SF-36 [62].

The neuropsychological test battery will consist of the following validated assessments: the Verbal Learning Memory Test (VLMT) [63], the Digit Span Backwards from the Wechsler Memory Scale-Revised (WMS-R) [64], the Trail Making Test (TMT) Parts A and B [65], the d2 Test of Attention [66], and the Regensburger Verbal Fluency Test (RWT) [67]. The VLMT evaluates verbal memory, including delayed free recall (the retention of learned words over time) and recognition ability. The Digit Span Backwards subtest from the WMS-R measures working memory. The TMT assesses visuomotor processing speed, attention, and executive functions such as mental flexibility. The d2 Test of Attention measures selective attention and processing speed. The RWT evaluates both formal lexical (phonemic) fluency and semantic fluency, which are indicators of divergent thinking.

#### Participant timeline

The schedule of the study including enrollment, intervention, and data assessment is given in Fig. 1 and Table 1.

#### Data collection, data management, and data storage

Study data will be collected using Heartbeat Medical [68] and REDCap (Research Electronic Data Capture) [69, 70]. Data collection will take place within the clinic and will comply with the legal data protection conditions of the EU GDPR (European Union General Data Protection Regulation). The links for the questionnaires will be personalized, with a password sent by post to activate the link. To obtain the most complete data possible, patients are reminded twice to complete the questionnaires. Data will only be stored and processed in encrypted form. Each participant will be given a research number, so it will not be possible for outsiders to link the data to the individual. Only those responsible for the study, who are bound by medical confidentiality, will have access to a list of personal data and corresponding research numbers. This list will be kept under lock and key. The data will be published anonymously.

#### Sample size calculation and statistical methods

Sample size calculation was performed with G*Power Version 3.1.9.7 [71, 72]. The sample size calculation is based on testing the primary hypothesis that inpatient psychosomatic treatment leads to a significantly lower mean PCS Score across the first two follow-up assessments (T1 and T2) as compared to treatment as usual. Because of the novelty of PASC, data on our primary outcome, the PCS Score, were not available in this population over different time points. Therefore, we assumed a conservative correlation of 0.5 among repeated measurements and a medium effect size. Based on a medium effect size (f = 0.25) and a power of 85%, a sample size of 98 participants is required, with 49 participants per group. Assuming a conservative dropout of 20%, we will randomize 118 participants, 58 for each condition.

We will perform standard techniques of data preparation with tests of normality and homogeneity and with imputation of missing data in the case of dropouts. Furthermore, we will conduct different descriptive analyses of sociodemographic and medical data. The primary statistical analyses will be a two-way mixed-design Analysis of Variance (ANOVA) with the independent variables being condition (treatment vs. treatment as usual) and time (T0, T1) and condition by time interaction as fixed effects as well as repeated measures ANOVA for the follow-up measurement with repeated measurements. Additionally, we will perform regression analyses to identify potential predictors of treatment outcomes. In all analyses, two-sided p values of < 0.05 are considered to indicate statistical significance. All analyses will be conducted using the statistical software SPSS.

#### Ethical aspects

We do not believe the trial raises any ethical concerns. We are not aware of any specific risks or disadvantages that may affect patients during the trial, and we do not expect any specific adverse or serious adverse events. Any unexpected adverse or serious adverse events will be documented. During the trial, patients will have access to medical advice or face-to-face contact with a member of the study team (psychologist or physician) at any time, if needed. Side effects of evidence-based psychotherapies are fortunately quite rare. However, potential adverse effects may include lack of treatment response, the emergence of new symptoms, worsening of existing symptoms, strains in the therapist-patient relationship, challenges or changes in work, family, or other social relationships, stigmatization, and the development of a pathological dependence on the therapist [73–75]. In the event of adverse effects, the psychotherapist, physician, or therapist, possibly in consultation with supervisors, will assess the need for further treatment. To prevent adverse events, several measures are implemented, including (i) a training program to raise awareness of adverse events, (ii) special training for physicians, psychologists, and other therapists for the treatment of PASC, and (iii) supervision of psychotherapists, physicians, and other therapists.

The Ethics Committee of the Medical Faculty of Friedrich-Alexander Universität Erlangen-Nürnberg (FAU) has approved the study (22–443_1-B). Written informed consent is required for all patients to participate and will be obtained after proof of verbal and written information and before group allocation. All participating patients can withdraw at any time without disadvantage. In the event of non-consent to participation in the study or withdrawal, patients will not suffer any disadvantages in terms of diagnostics, psychosomatic, or further treatment. Patients will be informed of this in detail in advance.

## Discussion

This non-randomized controlled study addresses the critical need for effective treatment strategies for PASC with comorbid mental illnesses. To the best of our knowledge, this is the first controlled study to examine the effectiveness of inpatient psychotherapy in PASC patients with comorbid mental illness. Existing evidence suggests that psychotherapeutic interventions can alleviate both mental health symptoms and physical complaints associated with persistent somatic symptoms [76, 77] and chronic illness [78–80], but their adaption to PASC requires further validation.

In Germany, inpatient psychotherapy is a prevalent approach for treating persistent somatic symptoms, particularly in patients with somatic symptom disorder and mental illnesses stemming from difficulties in coping with chronic illness (e.g., cancer, post-transplantation). Both conditions are relevant in PASC, making inpatient psychotherapy a suitable intervention. Treatment protocols for somatic symptom disorder often incorporate interventions of cognitive behavioral therapy (CBT) [81] and psychodynamic short-term therapies [82, 83], both of which are validated in numerous randomized control trials. These interventions can largely be adopted for the treatment of PASC. However, there is one important aspect in which the treatment of PASC differs from the treatment of somatoform disorders. While in inpatient psychotherapy for the treatment of somatoform disorders medical treatments and physical diagnostics are minimized to focus on the psychological context, the focus on the body is an important therapeutic element in PASC, which is primarily used in the concept of pacing. To prevent post-exertional malaise, patients should learn to listen to their body’s signals and better recognize their limits so as not to exceed their resources. Therefore, the treatment of PASC should integrate both physical and psychological factors, acknowledging their combined role in symptom development and maintenance and offering a holistic approach to patient recovery.

If the intervention is shown to be effective in reducing PASC symptoms and improving mental illness, we expect that direct and indirect costs related to PASC will be reduced, particularly in comorbid mental illnesses, which often require extensive and prolonged medical care [84, 85]. Moreover, effective psychotherapeutic treatment could potentially reduce the burden on healthcare systems by improving patients’ ability to manage their symptoms on their own and reducing the excessive use of healthcare services. In addition to that, our treatment can enhance patients’ overall quality of life, daily functioning, and social participation by alleviating symptoms of anxiety, depression, fatigue, and neurocognitive impairments.

In case the intervention is shown to be effective, implementation of the treatment of PASC in inpatient psychotherapy could have a large public health relevance. Furthermore, the findings of this study could have significant implications for the outpatient psychotherapeutic treatment of PASC. The success of the intervention could lead to the development of tailored outpatient programs, which would be more accessible to a larger number of patients. This transition could enhance the continuity of care and provide ongoing support for PASC patients, fostering long-term recovery and symptom management.

### Strengths and limitations

The study’s strengths lie in its robust control design and comprehensive assessment tools. The use of a control group allows for a comparison of outcomes between patients receiving the intervention and those receiving treatment as usual, providing a clearer understanding of the intervention’s effectiveness. Furthermore, the extensive neurocognitive test battery and the detailed measurement of patient-reported outcomes ensure that a wide range of symptoms and impacts are captured. This comprehensive approach allows for a thorough evaluation of the intervention’s effects on both the physical and mental health aspects of PASC. The prior comprehensive interdisciplinary diagnostics in our Post-COVID Center ensure that participants are adequately diagnosed with PASC and have a significant level of psychological distress, making them suitable candidates for psychosomatic treatment.

Despite these promising aspects of this study, several limitations must be considered. One significant limitation is the non-randomized allocation of patients to the control group, which is based on patients’ choice. This self-selection could introduce bias, as those who choose to participate in the intervention may have different levels of motivation or severity of symptoms compared to those who do not. This could potentially affect the generalizability of the study’s findings. Additionally, the study’s reliance on self-reported measures might introduce response bias. Ensuring a large and diverse sample size, along with rigorous statistical analysis, will be crucial in mitigating these limitations.

In conclusion, this study protocol addresses a critical gap in the treatment of PASC syndrome with comorbid mental illnesses. The potential benefits for both health economic factors and patient quality of life are substantial. While limitations such as the non-randomized control group allocation must be acknowledged, the study’s strengths, including its robust design and comprehensive outcome measurements, provide a solid foundation for evaluating the effectiveness of inpatient psychosomatic treatment for PASC. The findings could have significant implications for the broader application of psychotherapeutic interventions in both inpatient and outpatient settings, ultimately contributing to improved care and outcomes for PASC patients.

## Electronic supplementary material

Below is the link to the electronic supplementary material.

Supplementary Material 1

## Data Availability

No datasets were generated or analysed during the current study.

## Abbreviations

FAU Friedrich: Alexander Universität Erlangen-Nürnberg
PASC Post: Acute Sequelae of COVID-19
WHO: World Health Organization
PTSD: Post-traumatic stress disorder
PEM: Post-exertional malaise
TAU: Treatment as usual
PCS Score: Post-COVID Score
PHQ-15: Patient Health Questionnaire-15
PHQ-9: Patient Health Questionnaire-9
GAD-7: Generalized Anxiety Disorder Scale – 7
ISI: Insomnia Severity Index
FSS: Fatigue Severity Scale
ESSI: Experienced Social Support Index
SOC-3: Sense of Coherence Scale-3
PSS-4: Perceived Stress Scale-4
SSD-12: Somatic Symptom Disorder Scale-12
PUK-2: Patient Competence in Coping with Cancer Questionnaire adapted for PASC symptoms
SF-36: Short Form Health Survey Questionnaire
WMS-R: Wechsler Memory Scale-R
VLMT: Verbal Learning and Memory Test
TMT: Trail Making Test
d2-R: Attention and Concentration Test
RWT: Regensburger Wortflüssigkeits-Test
PCR: Polymerase Chain Reaction
SPIRIT: Standard Protocol Items: Recommendation for Interventional Trials
ICD-10: International Classification of Diseases, 10th Revision
CBT: Cognitive-Behavioral therapy
ACT: Acceptance and Commitment therapy
REDCap: Research Electronic Data Capture
EU GDPR: European Union General Data Protection Regulation
ANOVA: Analysis of Variance
SPSS: Statistical Package for the Social Sciences
